# Molecular Characterization and Phylogenetic Analysis of *Spirometra* Tapeworms from Snakes in Hunan Province

**DOI:** 10.3390/vetsci9020062

**Published:** 2022-02-01

**Authors:** Shu-Yu Chen, Teng-Fang Gong, Jun-Lin He, Fen Li, Wen-Chao Li, Li-Xing Xie, Xin-Rui Xie, Yi-Song Liu, Ying-Fang Zhou, Wei Liu

**Affiliations:** 1 Research Center for Parasites & Vectors, College of Veterinary Medicine, Hunan Agricultural University, Changsha 410128, China; ShuyuChen2021@stu.hunau.edu.cn (S.-Y.C.); GongTF@stu.hunau.edu.cn (T.-F.G.); hejunlin607@163.com (J.-L.H.); loislf@163.com (F.L.); Leo0725@stu.hunau.edu.cn (W.-C.L.); 1981318528@stu.hunau.edu.cn (X.-R.X.); liuyisong@hunau.edu.cn (Y.-S.L.); 2 Orient Science & Technology College, Hunan Agriculture University, Changsha 410128, China; xlx5652397@163.com; 3 Hunan Provincial the Key Laboratory of Protein Engineering in Animal Vaccine, College of Veterinary Medicine, Hunan Agricultural University, Changsha 410128, China

**Keywords:** genetic variation, phylogenetic analysis, ribosomal DNA, *Spirometra erinaceieuropaei*

## Abstract

Sparganosis is a neglected zoonotic parasitic disease that poses huge threats to humans worldwide. Snakes play an important role in sparganosis transmission because they are the most common second intermediate hosts for *Spirometra* parasites. However, the population genetics of *Spirometra* isolates from snakes is currently not well studied in China. The present study was performed to explore the molecular characteristics and phylogenetic analysis of *Spirometra* tapeworms from different species of snakes in Hunan Province. This study obtained 49 *Spirometra* isolates from 15 geographical areas in Hunan Province, Central China. Subsequently, the 18S and 28S ribosomal DNA (rDNA) fragments were amplified from the isolated parasites, and their sequences were analyzed to assess their genetic diversity. Phylogenetic analyses were performed using the maximum likelihood algorithm. The results showed that sequence variations among these isolates were 0–2.3% and 0–0.1% for 18S and 28S rDNA, respectively. The phylogenetic analysis showed that all *Spirometra* isolates from Hunan Province were clustered into the same branch with *Spirometra erinaceieuropaei* isolated from other areas (China, Vietnam, Australia). Moreover, the phylogenetic trees revealed that *Spirometra* is closely related to *Adenocephalus*, *Pyramicocephalus*, *Ligula*, *Dibothriocephalus*, *Schistocephalus*, and *Diphyllobothrium*. The *Spirometra* isolates of different hosts/regions in Hunan Province are not host segregated or geographically isolated, and support for the taxonomic status of *Spirometra* tapeworms in China has been added. These results provide reference values for future accurate identification and taxonomic status of *Spirometra* tapeworms in China.

## 1. Introduction

Human sparganosis is a worldwide disease caused by the larva (sparganum) of the genus *Spirometra* [1,2]. Humans can be infected through eating undercooked frog or snake meat and drinking polluted water [3,4]. Although sparganum has been reported to commonly reside in subcutaneous tissues and muscles, they can also migrate to the abdominal cavity, internal organs, eyes, and brain, which can form masses or space-occupying lesions in the body that cause local tissue damage and paralysis [5,6].

More than 10 species of the genus *Spirometra* have been reported, of which *Spirometra erinaceieuropaei* mainly infects humans. The first reported human case of sparganosis was discovered in 1882 by Patrick Manson from a man’s autopsy in Xiamen, and was named *Ligula mansoni* a year later [7]. *Sparganosis* has been mainly reported in China and can also be found in Europe (Poland, Italy, France, and the Czech Republic), Asia (Korea, Japan, Thailand, and Laos), South America (Ecuador, Paraguay, and Venezuela), and North America [5,8,9]. The reason for the high infection rate in China is mainly related to local customs. Superstitious people stick raw frog or snake flesh on skin wounds and even swallow tadpoles or snake bile in remote regions of China [4,10,11]. Another reason is the high infection rate of frogs and snakes in China. A survey showed that 14.3% (31/217) and 91.7% (344/375) of frogs and snakes, respectively, were infected in Hunan Province [11,12].

Although an important genus in zoonosis, the taxonomy of the *Spirometra* species has been controversial for a long time. It has also been suggested in some studies that the genus *Spirometra* belongs to the genus *Diphyllobothrium*, and should not form a separate genus [13,14]. Meanwhile, the valid species of *Spirometra* has also been unclear. This is still a mystery whether the pathogen of Chinese sparganosis is *S. erinaceieuropaei*, *Spirometra decipiens*, or both [11]. In the recent study of Yamasaki, it was found that two *Spirometra* species in Asia, neither of which is close to likely *S. erinaceieuropaei* originating from Poland, and lineage Type I is genetically diverse and widely distributed, however Type II is known so far only from Japan and Korea [15]. The primary and secondary ribosomal DNA (rDNA) structures remain stable during the long evolutionary process, which is one of the tools for studying phylogenetic evolution in parasites [16]. In the last few years of studies, ITS, 16S rDNA, 18S rDNA, and 28S rDNA have been used to establish the phylogenetic relationship of *Taenia* species [9,17,18,19,20,21]. The 18S and 28S rDNA contain both variable and conserved regions, which make them handy molecular markers to solve phylogenetic relationships at different levels [22]. This study analyzed the genetic diversity of the 18S and 28S rDNA sequences of *Spirometra* isolates from seven different hosts in 15 geographical regions in Hunan Province, and constructed the *Diphyllobothriidae* evolutionary tree. The main objectives of this study were as follows: (1) describe sample morphology; (2) perform a genetic diversity analysis of the collected isolates from different geographical locations and hosts in Hunan Province, China; and (3) investigate the taxonomic status of *Spirometra* isolates using 18S and 28S rDNA sequences from snakes in Hunan Province.

## 2. Materials and Methods

### 2.1. Sample Collection

**Figure 1:**
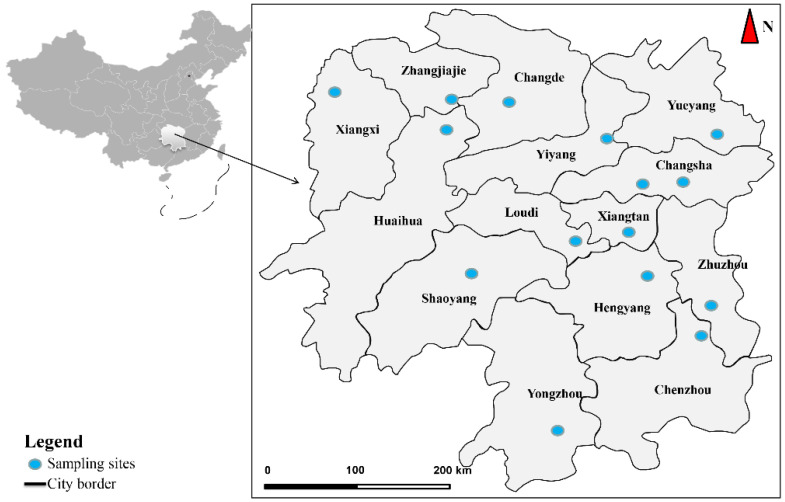
The sampling sites of *Spirometra* isolates in Hunan Province.

This study collected 49 samples from the field site in 15 geographical locations of Hunan Province in Southern China between April and September 2018 (Table 1). Figure 1 provides a scheme of the geographical locations of the collected *Spirometra* tapeworms. *Spirometra* tapeworms were isolated from muscles and subcutaneous tissues of three snake species of the family *Colubridae*, i.e., *Ptyas dhumnades* (Cantor, 1842), *Elaphe carinata* (Günther, 1864), and *Elaphe taeniura* (Cope, 1861), as well as from the intestines of the family *Felidae*, i.e., *Panthera tigris* (Linnaeus, 1758), *Prionailurus bengalensis* (Kerr, 1792), *Felis silvestris* (Schreber, 1777), and feral domestic cats. The collected samples were then fixed in 70% ethanol and kept at −20 °C for the molecular analysis.

### 2.2. Morphological Observations

The live worms were washed by water three times, and then sprayed with heavy metal on the surface. The morphology was made using the SEM-6380LV scanning electron microscope (JEOL, Akishima, Japan). The scolex of the sparganum and the scolex, gravid proglottid, and egg of *Spirometra* tapeworms were directly glued to the sample table and sprayed with a gold coating, and photographs were taken using a JSM-6380LV scanning electron microscope.

### 2.3. DNA Extraction and Enzymatic Amplification

The total genomic DNA was extracted from individual samples using the Wizard^®^ SV Genomic DNA Purification System (Promega Corporation, Madison, WI, USA) following the manufacturer’s protocol. Two ribosome markers (18S and 28S rDNA) were amplified by polymerase chain reaction (PCR) using the primer combinations listed in Appendix A. PCR reactions were carried out in a 25 μL reaction mixture containing 8.5 μL distilled water, 12.5 μL Taq PCR Master Mix (Thermo Fisher Scientific, Waltham, MA, USA), 1 μL of each primer (25 pmol/L), and 2 μL DNA template in a thermal cycler (Biometra, Göttingen, Germany). For the 18S rDNA, the steps were 94 °C for 5 min (first denaturation) and five cycles of 96 °C for 1 min, 44 °C for 1 min, and 72 °C for 2 min, followed by 25 cycles with annealing temperature increased to 48 °C and then by 5 min at 72 °C (final extension). For the 28S rDNA, the steps were 94 °C for 5 min and 35 periods of 94 °C for 30 s, 50 °C for 30 s, and 72 °C for 1 min, followed by 72 °C for 5 min. A negative sample (no DNA) was used in each amplification run. Positive PCR products were purified and then sequenced in both directions by the Tsingke Company (Changsha, China).

### 2.4. Sequence Analysis

The obtained sequences in this study and the reference sequences were aligned using Clustal X 1.7 software [23]. The DAMBE v.5.2 program was used to measure the nucleotide substitution saturation [24]. In addition, the obtained sequences in this research were also compared with *S. erinaceieuropaei* isolates from Australia (*Canis familiaris*), Vietnam (*Xenochrophis flavipunctatus*), and China (*Amphiesma stolatum* and *Rana nigromaculata*) for 18S rDNA sequences, and Australia (*C. familiaris*), Vietnam (*X. flavipunctatus*), and China (*A. stolatum*) for 28S rDNA sequences, using the Megalign procedure in DNASTAR 5.0 software [25]. Moreover, DnaSP 5.0 was used to analyze the diversity indices (nucleotide diversity (π) and haplotype diversity (Hd)) of these three gene sequences obtained in the current research [26].

### 2.5. Phylogenetic Analysis

All of the sequences are aligned using Clustal W in MAGE7.0. The best nucleotide substitution models were selected using JModelTest0.1. Phylogeny was estimated using a maximum likelihood algorithm (ML) in MEGA7.0. The stability of the tree was calculated based on 1000 bootstrap replicates. Genetic relationships with other *Diphyllobothriidae* species as in-group and *Bothriocotyle solinosomum* as out-group were evaluated (Appendix B).

## 3. Results

### 3.1. Morphological Characteristics

**Figure 2:**
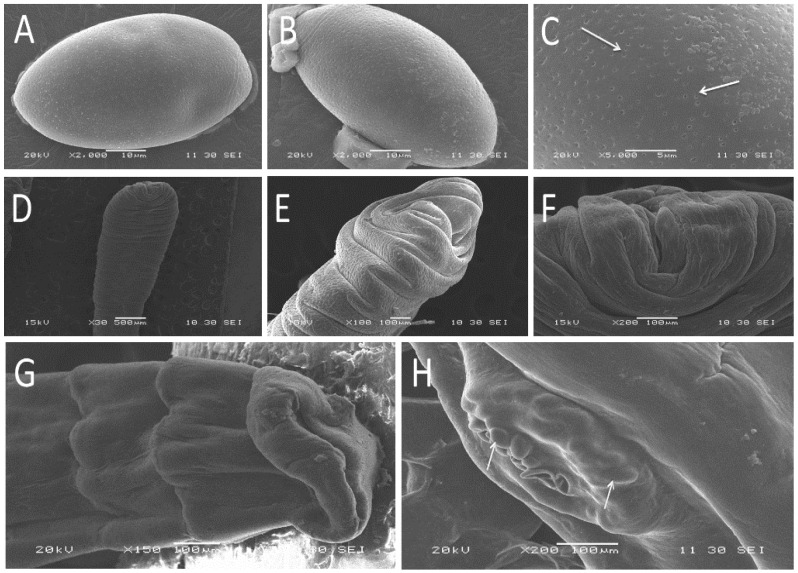
Scanning electron micrographs of *Spirometra* tapeworms collected from different hosts in Hunan Province, China. Egg (**A**,**B**). Detail of egg surface filled with pores (**C**). The scolex of larva, front view (**D**) and lateral view (**E**). Detail view of scolex (**F**). The scolex of adult (**G**). Detail view of egg in utero at the gravid proglottids (**H**).

In the scanning electron microscope study, the egg of *Spirometra* tapeworms was olive-shaped with slightly pointed ends and a slightly raised side, filled with many pores on the surface (Figure 2A–C). The scolex of the sparganum was flat, unsegmented, and with a wide front end, horizontal stripes, and apparent depression in the middle of the top end (Figure 2D–F). The adults were flat and segmented. The top of the adult scolex was sunken inward, and without other structure (Figure 2G). Moreover, many eggs existed in utero at the gravid proglottids (Figure 2H).

### 3.2. Genetic Characterisations of Spirometra Tapeworms

In this study, 49 and 49 PCR amplicons from 49 isolated samples were successfully amplified for 18S and 28S rDNA, respectively. No size differences were observed for any rDNA region among the amplicons tested (data not shown). The deletions and alignment lengths of the 18S and 28S rDNA were 2006–2010 and 1014 bp, respectively. The 28S rDNA target fragment amplified in this study is the front part of the entire 28S gene (highly protected area).

This study analyzed 49 18S sequences of *Spirometra* isolates. Intraspecific nucleotide variations within all isolates obtained in the present study were 0–2.3%. However, the 18S sequences obtained in the current study showed lower nucleotide variations of 0–1.6% compared with those of *S. erinaceieuropaei* from GenBank (China (KX528089 and HQ228991), Vietnam (KY552802), and Australia (KY552801). The pairwise comparison of the 28S rDNA sequences in the present paper showed 0–0.1% nucleotide variations. The sequence variation analysis for the 28S rDNA sequences showed higher nucleotide variations of 0–0.2% compared with those of *S. erinaceieuropaei* from GenBank (China (HQ228992), Vietnam (KY552835), and Australia (KY552836), and 0.60-0.90% compared with Diphyllobothriidea tapeworms (*Schistocephalus solidus*, *Diphyllobothrium scoticum*, *Diphyllobothrium sprakeri*, *Diphyllobothrium tetrapterum*, *Diphyllobothrium lanceolatum*, *Diphyllobothrium cordatum*, *Pyramicocephalus phocarum*, *Adenocephalus pacificus*, and *Ligula pavlovskii*).

The amplified 18S gene fragment sequence was 2006–2010 bp in length with 18 polymorphic sites. Moreover, insertions or deletions were found within the amplified fragments. Table 2 shows that the nucleotide diversity of the 18S sequences was 0.00062, which defined eight haplotypes with a haplotype diversity of 0.392. For 28S rDNA sequences (1014 bp), one polymorphic site was detected among 49 specimens examined in the present study, with no insertion or deletion. The diversity indices are shown in Table 2. The nucleotide diversity for the 28S rDNA sequences was 0.00021, defining two haplotypes with a haplotype diversity of 0.215.

### 3.3. Phylogenetic Relationship of S. erinaceieuropaei

**Figure 3:**
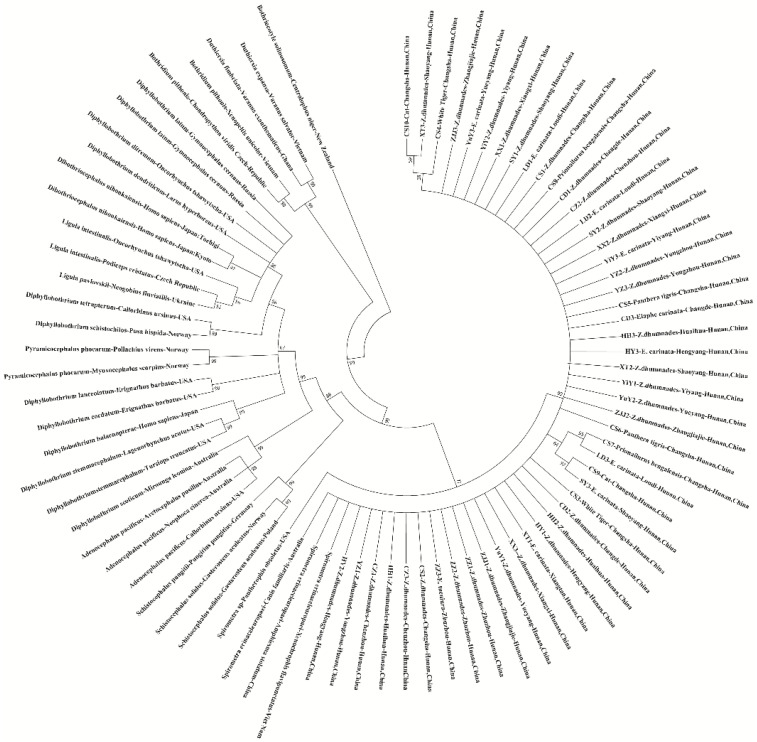
Maximum likelihood estimates of the phylogenetic relationships of *Spirometra* tapeworms based on 18S and 28S sequences computed in MEGA version 7.0.26 under the GTR model. The confidence levels in each node were assessed with the boot-strap method (1000 pseudo replicates) and bootstrap values >50.

A phylogenetic tree based on the 18S and 28S sequences was constructed using the ML method under the general time-reversible (GTR) model by MEGA7.0 (Figure 3). Data showed that all the isolated samples recorded in this study were grouped into one group, and clustered into the same branch with the *S. erinaceieuropaei* in Genbank from other countries (China, Vietnam, and Australia). In addition, a relatively complete phylogenetic *Diphyllobothriidae* tree was constructed based on the 18S and 28S sequences. In the current study, *Spirometra* spp. formed a separate group and were closely related to *Schistocephalus* spp. Moreover, the genus *Diphyllobothrium* occupied most of the phylogenetic tree, which was made up of *Adenocephalus* spp., *Pyramicocephalus* spp., *Ligula* spp., *Dibothriocephalus* spp., and *Schistocephalus* spp. However, the relationships among the species of *Diphyllobothrium* by 18S and 28S sequence were not established. *Duthiersia fimbriata* and *Duthiersia expansa* formed the *Duthiersia* spp. branch and then formed a sister group, the *Bothridium pithonis* branch.

## 4. Discussion

The species classification of *Spirometra* has been controversial. For many years, many researchers considered *S. erinaceieuropaei* as a global species [5,15]. As more and more mitochondrial gene sequences of *S. erinaceieuropaei* have been reported globally in recent years, studies have found that *S. erinaceieuropaei* in China and Southeast Asia and *S. erinaceieuropaei* in Europe do not belong to the same branch, which also means that the Chinese and Southeast Asia region may not be the previously thought *S. erinaceieuropaei* [7]. The present study aimed to analyze the genetic diversity of *Spirometra* tapeworms from snakes and to explore the taxonomic status of *Spirometra* isolates from Hunan Province on a molecular level. At the same time, this study provides the description of the morphology of *Spirometra* isolates from snakes in Hunan Province based on scanning electron microscopy, which will lay the foundation for future *Spirometra* tapeworm species classification in China.

The study used 18S and 2S rDNA genes to explore the intraspecific nucleotide variations of the *Spirometra* isolates in Hunan Province, China. The results show that the maximum variation values for the 18S and 28S rDNA sequences were 0–2.3% and 0–0.1%, respectively, among the *Spirometra* isolates from different hosts examined (*Zaocys dhumnades*, *Elaphe carinata*, *Elaphe taeniura*, *Panthera tigris*, *Prionailurus bengalensis*, *Felis silvestris*, and cat). The sequence variation analysis for the 18S gene showed 0–2.3% nucleotide divergence compared with those of *S. erinaceieuropaei* in China (*R. nigromaculata* KX528089 and *A. stolatum* HQ228991), Vietnam (*X. flavipunctatus* KY552802), and Australia (*C. familiaris* KY552801). This suggests that both host specificity and geographical effects are not the main factors contributing to the genetic variation of *S. erinaceieuropaei*, which can also be based on the results of the sequence variation analysis of 28S rDNA. This conclusion is in accordance with recently conducted research [9,21,27].

Haplotype and nucleotide diversities are two important indicators to measure the genetic variation of a gene. A base change can form a haploid type, and haploid type diversity can rapidly rise in a concise time. However, nucleotide base changes have little effect on nucleotide diversity. The rise of nucleotide diversity needs a long accumulation time. Thus, nucleotide diversity is more applicable for measuring the genetic diversity of a species [28]. For most organisms, a nucleotide diversity of >0.01 is considered a large variation [29]. In the current study, the nucleotide diversity of 18S and 28S rDNA genes of the *Spirometra* isolates was 0.00062 and 0.00028, respectively, which was lower than 0.01. The results showed that the genetic variation of *Spirometra* isolates from different hosts in Hunan Province was low.

In recent years, it has been shown by the molecular genetic evolution analysis that China and Poland are in different branches. Some scholars have proposed that *Spirometra* tapeworms should be restored to the title of *Spirometra mansoni* in China and Southeast Asia [7,15,30]. The phylogenetic tree based on 18S and 28S sequences showed that all the *Spirometra* isolates from different regions in Hunan Province formed a branch with *S. erinaceieuropaei* from Genbank from other countries (China, Vietnam, and Australia), except for the *S. erinaceieuropaei* reported in the United States. This result is consistent with Kuchta et al.’s proposal that China and Southeast Asia should be classified as *S. mansoni*, North America should be classified as S. decipiens, and Europe should be classified as *S. erinaceieuropai*. In the current study, phylogenetic trees revealed that *Spirometra* is closely related to *Adenocephalus*, *Pyramicocephalus*, *Ligula*, *Dibothriocephalus*, *Schistocephalus*, and *Diphyllobothrium* and forms a branch, which is similar to the study of Waeschenbach and Hernandez [18,21].

## 5. Conclusions

In our study, the genetic variability among different distinct developmental stages (larvae and adults) of *Spirometra* tapeworms isolated from 15 geographical areas in Hunan Province was analyzed for the 18S and 28S rDNA genes. The results revealed genetic variability in 18S and 28S rDNA. The phylogenetic tree based on 18S and 28S sequences revealed that the *Spirometra* isolates of different hosts/regions in Hunan Province are not host segregated or geographically isolated, and support for the taxonomic status of *Spirometra* tapeworms in China was thus added. These results provide reference values for future accurate identification and taxonomic status of *Spirometra* tapeworms in China.

## Figures and Tables

**Table 1:** Geographical origins (different locations in Hunan Province, China) of *Spirometra* tapeworms isolates used in this study, as well as their GenBank accession numbers for the 18S and 28S sequences.

Geographical Origins	Host	Location	Sample Codes
Yiyang City			
Lanxi Town, Heshan District	*Zaocys dhumnades*	112°46′ E, 28°59′ N	HuN-YiY1
	*Z. dhumnades*	112°46′ E, 28°59′ N	HuN-YiY2
	*Elaphe carinata*	112°46′ E, 28°59′ N	HuN-YiY3
Changde City			
Taizimiao Town, Hanshou County	*Z. dhumnades*	111°96′ E, 28°77′ N	HuN-CD1
	*Z. dhumnades*	111°96′ E, 28°77′ N	HuN-CD2
	*E. carinata*	111°96′ E, 28°77′ N	HuN-CD3
Yongzhou City			
Taiping Town, Ningyuan County	*Z. dhumnades*	112°13′ E, 25°67′ N	HuN-YZ1
	*Z. dhumnades*	112°13′ E, 25°67′ N	HuN-YZ2
	*Z. dhumnades*	112°13′ E, 25°67′ N	HuN-YZ3
Hengyang City			
Xuanzhou Town, Hengyang County	*Z. dhumnades*	112°85′ E, 27°24′ N	HuN-HY1
	*Z. dhumnades*	112°85′ E, 27°24′ N	HuN-HY2
	*E. carinata*	112°85′ E, 27°24′ N	HuN-HY3
Xiangtan City			
Jinshi Country, Xiangtan County	*Z. dhumnades*	112°75′ E, 27°59′ N	HuN-XT1
	*Z. dhumnades*	112°75′ E, 27°59′ N	HuN-XT2
	*E. carinata*	112°75′ E, 27°59′ N	HuN-XT3
Shaoyang City			
Shizhu Town, Dongkou County	*Z. dhumnades*	110°73′ E, 27°25′ N	HuN-SY1
	*Z. dhumnades*	110°73′ E, 27°25′ N	HuN-SY2
	*E. carinata*	110°73′ E, 27°25′ N	HuN-SY3
Zhuzhou City			
Jieshou Town, Chaling County	*Z. dhumnades*	113°43′ E, 26°61′N	HuN-ZZ1
	*Z. dhumnades*	113°43′ E, 26°61′N	HuN-ZZ2
	*Elaphe taeniura*	113°43′ E, 26°61′N	HuN-ZZ3
Changsha City			
Langli Town, Changsha County	*Z. dhumnades*	113°13′ E, 28°19′ N	HuN-CS1
	*Z. dhumnades*	113°13′ E, 28°19′ N	HuN-CS2
Changsha Ecological Zoo, Tianxin District	*White Tiger*	113°01′ E, 28°04′ N	HuN-CS3
	*W. Tiger*	113°01′ E, 28°04′ N	HuN-CS4
	*Panthera tigris*	113°01′ E, 28°04′ N	HuN-CS5
	*P. tigris*	113°01′ E, 28°04′ N	HuN-CS6
	*Prionailurus bengalensis*	113°01′ E, 28°04′ N	HuN-CS7
	*P.bengalensis*	113°01′ E, 28°04′ N	HuN-CS8
	*Cat*	113°01′ E, 28°04′ N	HuN-CS9
	*Cat*	113°01′ E, 28°04′ N	HuN-CS10
Loudi city			
Suoshi Town, Shuangfeng County	*E. carinata*	112°12′ E, 27°32′ N	HuN-LD1
	*E. carinata*	112°12′ E, 27°32′ N	HuN-LD2
	*E. carinata*	112°12′ E, 27°32′ N	HuN-LD3
Chenzhou City			
Longhai Town, Anren County	*Z. dhumnades*	113°29′ E, 26°48′ N	HuN-CZ1
	*Z. dhumnades*	113°29′ E, 26°48′ N	HuN-CZ2
	*Z. dhumnades*	113°29′ E, 26°48′ N	HuN-CZ3
Huaihua City			
Qijiaping Town, Yuanling County	*Z. dhumnades*	110°86′ E, 28°88′ N	HuN-HH1
	*Z. dhumnades*	110°86′ E, 28°88′ N	HuN-HH2
	*Z. dhumnades*	110°86′ E, 28°88′ N	HuN-HH3
Zhangjiajie City			
Dongxi Coutry, Cili County	*Z. dhumnades*	110°83′ E, 29°14′ N	HuN-ZZJ1
	*Z. dhumnades*	110°83′ E, 29°14′ N	HuN-ZZJ2
	*Z. dhumnades*	110°83′ E, 29°14′ N	HuN-ZZJ3
Yueyang City			
Tongshi Town, Pingjiang County	*Z. dhumnades*	113°72′ E, 28°75′ N	HuN-YuY1
	*Z. dhumnades*	113°72′ E, 28°75′ N	HuN-YuY2
	*E. taeniura*	113°72′ E, 28°75′ N	HuN-YuY3
Xiangxi City			
Xichehe Town, Longshan County	*Z. dhumnades*	109°54′ E, 29°09′ N	HuN-XX1
	*Z. dhumnades*	109°54′ E, 29°09′ N	HuN-XX2
	*Z. dhumnades*	109°54′ E, 29°09′ N	HuN-XX3

**Table 2:** Diversity indices for *Spirometra* tapeworms using nucleotide data of the ribosomal 18S rRNA (2006–2010 bp) and 28S rRNA (1013 bp) gene sequences obtained in the present paper.

	N	S	H	π	Hd	K
18S	49	18	8	0.00062	0.392	1.244
28S	49	2	3	0.00028	0.275	0.281

N: number of isolates; S: number of polymorphic sites; H: number of haplotypes; π: nucleotide diversity; Hd: haplotype (gene) diversity; K: average number of nucleotide differences.

## Data Availability

Please refer to suggested Data Availability Statements at https://www.ncbi.nlm.nih.gov/nuccore/?term=18S+and+Spirometra+erinaceieuropaei and https://www.ncbi.nlm.nih.gov/nuccore/?term=28S+and+Spirometra+erinaceieuropaei.

## Appendix A

**Table A1:** Primers used to amplify the sequences studied.

Gene	Name	Sequence (5′–3′)	References
18S	PL3F	ACCTGGTTGATCCTGCCAG	Barta et al., 1997
	PL3R	CTTCCGCTGGTTCACCTACGG	
28S	28S-F	TGATAGGTTATTTAAACTGGC	This study
	28S-R	ACCCGACCCGTCTTGAAACA	

## Appendix B

**Table A2:** *Spirometra* isolates included in the molecular analysis, and accession numbers of the corresponding individual sequence.

Species	Country of Origin	Host	Sample Codes	Accession Number		References
				18S	28S	
*Spirometra erinaceieuropaei*	Yiyang City, Hunan Province, China	*Zaocys dhumnades*	HuN-YiY1	MZ267595	MZ293029	This study
		*Z. dhumnades*	HuN-YiY2	MZ267596	MZ293030	This study
		*Elaphe carinata*	HuN-YiY3	MZ267597	MZ293031	This study
	Changde City, Hunan Province, China	*Z. dhumnades*	HuN-CD1	MZ267569	MZ293003	This study
		*Z. dhumnades*	HuN-CD2	MZ267570	MZ293004	This study
		*E. carinata*	HuN-CD3	MZ267571	MZ293005	This study
	Yongzhou City, Hunan Province, China	*Z. dhumnades*	HuN-YZ1	MZ267598	MZ293035	This study
		*Z. dhumnades*	HuN-YZ2	MZ267599	MZ293036	This study
		*Z. dhumnades*	HuN-YZ3	MZ267600	MZ293037	This study
	Hengyang City, Hunan Province, China	*Z. dhumnades*	HuN-HY1	MZ267583	MZ293017	This study
		*Z. dhumnades*	HuN-HY2	MZ267584	MZ293018	This study
		*E. carinata*	HuN-HY3	MZ267585	MZ293019	This study
	Xiangtan City, Hunan Province, China	*Z. dhumnades*	HuN-XT1	MZ267589	MZ293023	This study
		*Z. dhumnades*	HuN-XT2	MZ267590	MZ293024	This study
		*E. carinata*	HuN-XT3	MZ267591	MZ293025	This study
	Shaoyang City, Hunan Province, China	*Z. dhumnades*	HuN-SY1	MZ267586	MZ293020	This study
		*Z. dhumnades*	HuN-SY2	MZ267587	MZ293021	This study
		*E. carinata*	HuN-SY3	MZ267588	MZ293022	This study
	Zhuzhou City, Hunan Province, China	*Z. dhumnades*	HuN-ZZ1	MZ267604	MZ293041	This study
		*Z. dhumnades*	HuN-ZZ2	MZ267605	MZ293042	This study
		*E. taeniura*	HuN-ZZ3	MZ267606	MZ293043	This study
	Changsha City, Hunan Province, China	*Z. dhumnades*	HuN-CS1	MZ267572	MZ293006	This study
		*Z. dhumnades*	HuN-CS2	MZ267573	MZ293007	This study
		*White Tiger*	HuN- CS3	MZ267607	MZ292995	This study
		*W. Tiger*	HuN- CS4	MZ267608	MZ292996	This study
		*Panthera tigris*	HuN- CS5	MZ267609	MZ292997	This study
		*P. tigris*	HuN- CS6	MZ267610	MZ292998	This study
		*Prionailurus bengalensis*	HuN- CS7	MZ267611	MZ292999	This study
		*P. bengalensis*	HuN- CS8	MZ267612	MZ293000	This study
		*Cat*	HuN- CS9	MZ267613	MZ293001	This study
		*Cat*	HuN- CS10	MZ267614	MZ293000	This study
	Loudi City, Hunan Province, China	*E. carinata*	HuN-LD1	MZ267580	MZ293014	This study
		*E. carinata*	HuN-LD2	MZ267581	MZ293015	This study
		*E. carinata*	HuN-LD3	MZ267582	MZ293016	This study
	Chenzhou City, Hunan Province, China	*Z. dhumnades*	HuN-CZ1	MZ267574	MZ293008	This study
		*Z. dhumnades*	HuN-CZ2	MZ267575	MZ293009	This study
		*Z. dhumnades*	HuN-CZ3	MZ267576	MZ293010	This study
	Huaihua City, Hunan Province, China	*Z. dhumnades*	HuN-HH1	MZ267577	MZ293011	This study
		*Z. dhumnades*	HuN-HH2	MZ267578	MZ293012	This study
		*Z. dhumnades*	HuN-HH3	MZ267579	MZ293013	This study
	Zhangjiajie City, Hunan Province, China	*Z. dhumnades*	HuN-ZZJ1	MZ267601	MZ293038	This study
		*Z. dhumnades*	HuN-ZZJ2	MZ267602	MZ293039	This study
		*Z. dhumnades*	HuN-ZZJ3	MZ267603	MZ293040	This study
	Yueyang City, Hunan Province, China	*Z. dhumnades*	HuN-YuY1	MZ267566	MZ293032	This study
		*Z. dhumnades*	HuN-YuY2	MZ267567	MZ293033	This study
		*E. taeniura*	HuN-YuY3	MZ267568	MZ293034	This study
	Xiangxi City, Hunan Province, China	*Z. dhumnades*	HuN-XX1	MZ267592	MZ293026	This study
		*Z. dhumnades*	HuN-XX2	MZ267593	MZ293027	This study
		*Z. dhumnades*	HuN-XX3	MZ267594	MZ293028	This study
	Guilin City, Guangxi Province, China	*Amphiesma stolatum*		HQ228991	HQ288992	Lee et al., 2010
	Xiangtan City, Hunan Province, China	*Rana nigromaculata*		KX528089		Zhang et al., 2017
	Australia	*Canis familiaris*		KY552801	KY552835	Kuchta et al., 2017
	Vietnam	*Xenochrophis flavipunctatus*		KY552802	KY552836	Kuchta et al., 2017
*Adenocephalus pacificus*	Australia	*Arctocephalus pusillus*		KY552774	KY552808	Kuchta et al., 2017
	USA	*Callorhinus ursinus*		KY552775	KY552810	Kuchta et al., 2017
	Australia	*Neophoca cinerea*		KY552776	KY552809	Kuchta et al., 2017
*Bothridium pithonis*	Czech Republic	*Chondropython viridis*		KY552803	KY552838	Kuchta et al., 2017
	Vietnam	*Xenopeltis unicolor*		KY552804	KY552839	Kuchta et al., 2017
*Dibothriocephalus nihonkaiensis*	Japan	*Homo sapiens*		AB512013	LC312467	Yanagida et al., 2021 Yamasaki et al., 2021
*Dibothriocephalus latus*	Russia	*Gymnocephalus cernuus*		DQ925309	DQ925326	Brabec et al., 2016
*Dibothriocephalus dendriticus*	USA	*Larus hyperboreus*		KY552779	KY552814	Kuchta et al., 2017
	United Kingdom	*Coregonus lavaretus*		KY552778	KY552812	Kuchta et al., 2017
*Dibothriocephalus ditremus*	United Kingdom	*Salvelinus alpinus*		KY552780	KY552813	Kuchta et al., 2017
	USA	*Oncorhynchus tshawytscha*		KY552787	KY552815	Kuchta et al., 2017
*Diphyllobothrium scoticum*	Australia	*Mirounga leonina*		KY552777	KY552811	Kuchta et al., 2017
*Diphyllobothrium dendriticum*	USA	*Larus hyperboreus*		KY552779	KY552814	Kuchta et al., 2017
*Diphyllobothrium schistochilos*	Norway	*Pusa hispida*		KY552782	KY552821	Kuchta et al., 2017
*Diphyllobothrium tetrapterum*	USA	*Callorhinus ursinus*		KY552786	KY552826	Kuchta et al., 2017
*Diphyllobothrium cordatum*	USA	*Erignathus barbatus*		KY552788	KY552882	Kuchta et al., 2017
*Diphyllobothrium lanceolatum*	USA	*Erignathus barbatus*		KY552789	KY552823	Kuchta et al., 2017
*Diphyllobothrium stemmacephalum*	USA	*Lagenorhynchus acutus*		AF124459	AF286943	Kuchta et al., 2017
*Diphyllobothrium balaenopterae*	Japan	*Homo sapiens*		KY552792	KY552824	Kuchta et al., 2017
*Duthiersia fimbriata*	Ghana	*Varanus exanthematicus*		AF267290	DQ925328	Kodedova et al., 2001 Brabec et al., 2006
*Duthiersia expansa*	Vietnam	*Varanus salvator*		KY552806	KY552840	
*Ligula intestinalis*	USA	*Oncorhynchus tshawytscha*		KY552783	KY552818	Kuchta et al., 2017
*Ligula intestinalis*	Czech Republic	*Podiceps cristatus*		KY552785	KY552819	Kuchta et al., 2017
*Ligula pavlovskii*	Ukraine	*Neogobius fluviatilis*		KY552784	KY552820	Kuchta et al., 2017
*Probothriocephalus alaini*	Atlantic Ocean	*Xenodermichthys copei*		KR780925	KR780881	Brabec et al., 2015
*Pyramicocephalus phocarum*	Norway	*Myoxocephalus scorpius*		KY552790	KY552827	Kuchta et al., 2017
	Norway	*Pollachius virens*		KY552791	KY552828	Kuchta et al., 2017
*Schistocephalus solidus*	Poland	*Gasterosteus aculeatus*		KY552797	KY552832	Kuchta et al., 2017
	Norway	*Gasterosteus aculeatus*		KY552798	KY552833	Kuchta et al., 2017
*Schistocephalus pungitii*	Germany	*Pungitius pungitius*		KY552799	KY552834	Kuchta et al., 2017
*Haplobothrium globuliforme*	Canada	*Amia calva*		AF124458	AF286926	Olson et al., 1999 Olson et al., 2001
